# Supplementary material for: Transcriptomic Analysis Reveals Key Roles of (p)ppGpp and DksA in Regulating Metabolism and Chemotaxis in Yersinia enterocolitica
Source: Int J Mol Sci. 2023 Apr 20;24(8):7612. doi: 10.3390/ijms24087612 (PMC10142893; doi:10.3390/ijms24087612)
Supplement: Supplementary file 1 [file ijms-24-07612-s001.zip › ijms-2325845-supplementary.pdf]

Supplementary Table S1. Primers used in this study.

Primers name	DNA Sequences (5' to 3')	Application
16s rRNA-F	GCACGTAATGGTGGGAACTC	qRT-PCR
16S rRNA-R	CTCCAATCCGGACTACGACA	qRT-PCR
YZ-*pdeR*-F	TTGCCAATAACATCCTGCTCTT	qRT-PCR
YZ -*pdeR*-R	GCCTATGCCGATACTGAATC	qRT-PCR
YZ -*yihX*-F	CCGAGTGGTGGTGCTATCTA	qRT-PCR
YZ -*yihX*-R	GATTCTGGCTTCAGGTTTGC	qRT-PCR
YZ -*rpsN*-F	ACCCGAACTTCCGCAAGA	qRT-PCR
YZ -*rpsN*-R	GTGTGAACTCATCCGACGAA	qRT-PCR
YZ -*rpoA*-F	CGGCAACTATCTTGGCTGAAC	qRT-PCR
YZ -*rpoA*-R	CAGTTAGCAGAGCGGACAGT	qRT-PCR
YZ -*trmD*-F	GGCAATCCAGCAATCCATCA	qRT-PCR
YZ -*trmD*-R	GGTCGGTACGAAGGAGTTGA	qRT-PCR
YZ -*dhaK*-F	AAGTCGCACTGATGTCTGGT	qRT-PCR
YZ -*dhaK*-R	GGCTTGACCGCATTCATACA	qRT-PCR
YZ -*gntT*-F	CCTCTCCTATGCCGAGTTCT	qRT-PCR
YZ -*gntT*-R	TGTGTCCATCACCGATTCCA	qRT-PCR
YZ -YE_RS15980-F	GCTTCCAACAAGTGCTATCAGA	qRT-PCR
YZ -YE_RS15980-R	CAAACATTTCTACCGCCACAAC	qRT-PCR
YZ -*dusB*-F	GTCTGCCGAACACCGTAACT	qRT-PCR
YZ -*dusB*-R	CGTATTCCGCCTCGCCATTA	qRT-PCR
YZ -*corA*-F	GGTAACTCAACGGTGGCATT	qRT-PCR
YZ -*corA*-R	GTCCAGCAGCAACTCATAGG	qRT-PCR
YZ -*epmA*-F	CGCAGCAATAATGGCAGAGA	qRT-PCR
YZ -*epmA*-R	AATGGATGTCCGTCACTGTC	qRT-PCR
YZ -*trmL*-F	CGGCTTACCCTCCAATGTTC	qRT-PCR
YZ -*trmL*-R	CAGGCTTCGTACACCACAAC	qRT-PCR
YZ -*fliE*-F	ACCAGCAAACAGCCCGTA	qRT-PCR
YZ -*fliE*-R	CGTCTTGATAAGCCGCTACTAA	qRT-PCR
YZ -*flgC*-F	GTTGATGTGACCGGCGAGAT	qRT-PCR
YZ -*flgC*-R	AGTGTCAGCGTCTTGAGCAT	qRT-PCR
YZ -*alaC*-F	GCCGTGGTGAAGACATCATT	qRT-PCR
YZ -*alaC*-R	CGAGGTTGAATAGCCGTGAG	qRT-PCR
YZ -*fyuA*-F	TGGCAGCAGCAGCATTATTC	qRT-PCR
YZ -*fyuA*-R	CATTGTCGGCATGTCGTAGG	qRT-PCR
YZ -*hypE*-F	CGCCACTACGCCATATTACC	qRT-PCR
YZ -*hypE*-R	TCCGCTTCGTTGATTTCCAT	qRT-PCR
YZ -*lsrD*-F	TGCCTGTTGTATGCCTTGAC	qRT-PCR
YZ -*lsrD*-R	GAGCCGTACCGAGAATGGA	qRT-PCR
YZ -*hisJ*-F	ACAAGGCTCGACTCAGGAAG	qRT-PCR
YZ -*hisJ*-R	CTCACTGCCAGCGACTTCAT	qRT-PCR
dksA -F	TAAGCCGCCGATCTGCTGAAT	To identify Δ*dksA*

dksA -R	GAAGTCAAGTCGGTTACCTT	To identify Δ*dksA*
relA -F	AACAGCAAATCCCCCAATTC	To identify Δ*relA*Δ*spoT* and Δ*dksA*Δ*relA*Δ*spoT*
relA -R	GGTGGCTCAGGCTATAGCAT	To identify Δ*relA*Δ*spoT* and Δ*dksA*Δ*relA*Δ*spoT*

Supplementary Table S2. Statistical analysis of RNA-Seq data in the WT and mutant strains.

Sample Name	Raw Reads	Raw Bases (bp)	Clean Reads	Clean Bases (bp)	Genome Mapped Reads	Genome Mapped Ratio (%)	Unique Mapped Reads	Unique Mapped Reads Ratio (%)
WT-1	23676006	3575076906	23309948	3050093381	22844598	98.00	21828056	93.64
WT-2	24703604	3730244204	24303912	3160428673	23818997	98.00	22818158	93.89
WT-3	23510642	3550106942	23131770	3056330141	22691768	98.10	21665010	93.66
Δ*dksA*-1	25354062	3828463362	24970570	3325072720	24401701	97.72	23580072	94.43
Δ*dksA*-2	28460700	4297565700	28065896	3752245167	27506717	98.01	26545712	94.58
Δ*dksA*-3	24358918	3678196618	24038218	3222763611	23548300	97.96	22576128	93.92
Δ*relA*Δ*spoT*-1	28023310	4231519810	27317700	3393662150	26738618	97.88	25971514	95.07
Δ*relA*Δ*spoT* -2	24525074	3703286174	24244748	3158094318	23764932	98.02	22970646	94.74
Δ*relA*Δ*spoT* -3	23932950	3613875450	23658478	3110151493	23193469	98.03	22583891	95.46
Δ*dksA*Δ*relA*Δ*spoT* -1	23258634	3512053734	22960764	3002781309	22519203	98.08	22025031	95.92
Δ*dksA*Δ*relA*Δ*spoT* -2	26148594	3948437694	25842156	3328877730	25365103	98.15	24723192	95.67
Δ*dksA*Δ*relA*Δ*spoT* -3	27424488	4141097688	27166778	3537542759	26584190	97.86	25769114	94.86

Supplementary Table S3. Statistical analysis of ribosome proteins biosynthesis in mutant strains compared to WT.

Gene ID	Gene Name	Description	FC Δ*dksA*/WT	*P* value Δ*dksA*/WT	FC Δ*relA*Δ*spoT* /WT	P value Δ*relA*Δ*spoT* /WT	FC Δ*dksA*Δ*relA*Δ*spoT* /WT	P value Δ*dksA*Δ*relA*Δ*spoT* /WT
YE_RS03125	*rpsT*	30S ribosomal protein S20	2.016	2.82E-05	5.674	4.32E-20	2.578	2.33E-08
YE_RS20925	*rpmH*	50S ribosomal protein L34	2.032	0.002101	6.358	3.43E-15	2.41	0.000259
YE_RS18770	*rplM*	50S ribosomal protein L13	2.937	3.23E-07	3.573	1.56E-12	3.147	3.23E-10
YE_RS04275	*rplS*	50S ribosomal protein L19	NS	NS	2.541	6.17E-13	NS	NS
YE_RS19585	*rpmD*	50S ribosomal protein L30	2.207	5.63E-08	2.587	5.10E-13	NS	NS
YE_RS18470	*rpsU*	30S ribosomal protein S21	2.204	0.008421	2.568	0.001224	2.063	0.011729
YE_RS19645	*rpsC*	30S ribosomal protein S3	2.027	1.79E-05	2.995	1.70E-10	NS	NS
YE_RS19635	*rpmC*	50S ribosomal protein L29	NS	NS	2.418	2.14E-08	NS	NS
YE_RS19630	*rpsQ*	30S ribosomal protein S17	NS	NS	2.044	0.000564	NS	NS
YE_RS00325	*rpmB*	50S ribosomal protein L28	2.311	0.000153	3.544	4.13E-08	2.03	0.002088
YE_RS00550	*rpmE*	50S ribosomal protein L31	NS	NS	3.809	5.32E-15	2.267	3.11E-08
YE_RS19545	*rplQ*	50S ribosomal protein L17	3.194	2.95E-12	3.114	4.28E-10	2.497	9.71E-08
YE_RS19605	*rpsH*	30S ribosomal protein S8	2.217	9.95E-08	4.844	6.50E-22	2.307	8.56E-10
YE_RS19600	*rplF*	50S ribosomal protein L6	2.403	9.81E-15	3.56	2.50E-24	NS	NS
YE_RS09610	*rplT*	50S ribosomal protein L20	2.037	2.60E-05	2.138	1.08E-05	NS	NS
YE_RS19650	*rplV*	50S ribosomal protein L22	2.123	2.12E-12	3.243	3.59E-17	NS	NS
YE_RS19655	*rpsS*	30S ribosomal protein S19	2.048	1.98E-07	2.416	4.02E-09	NS	NS
YE_RS01490	*rplL*	50S ribosomal protein L7/L12	NS	NS	2.68	1.29E-11	NS	NS

YE_RS02280	*rpsO*	30S ribosomal protein S15	2.4	2.19E-05	5.133	3.04E-12	2.292	0.000277
YE_RS02045	*rpsF*	30S ribosomal protein S6	2.523	1.73E-10	3.05	3.35E-09	2.406	8.76E-09
YE_RS16465	*rpsB*	30S ribosomal protein S2	2.505	1.10E-08	4.681	7.39E-17	2.774	6.66E-10
YE_RS08205	*rpmF*	50S ribosomal protein L32	NS	NS	2.705	6.74E-06	NS	NS
YE_RS19570	*rpmJ*	50S ribosomal protein L36	2.346	3.14E-05	2.86	2.01E-05	0.219	8.03E-05
YE_RS19615	*rplE*	50S ribosomal protein L5	2.199	9.20E-13	3.503	7.15E-19	NS	NS
YE_RS19610	*rpsN*	30S ribosomal protein S14	2.457	5.75E-11	5.006	1.75E-20	2.373	2.35E-09
YE_RS19680	*rpsJ*	30S ribosomal protein S10	3.235	1.45E-14	6.795	2.35E-36	3.962	4.46E-17
YE_RS02175	*rplU*	50S ribosomal protein L21	2.396	6.61E-05	2.945	3.85E-06	2.051	0.001674
YE_RS19665	*rplW*	50S ribosomal protein L23	2.458	3.58E-10	4.269	5.34E-27	2.432	9.85E-12
YE_RS19660	*rplB*	50S ribosomal protein L2	2.039	4.33E-07	3.18	3.34E-16	NS	NS
YE_RS01480	*rplA*	50S ribosomal protein L1	2.384	2.81E-10	4.441	6.94E-25	NS	NS
YE_RS01485	*rplJ*	50S ribosomal protein L10	NS	NS	3.823	5.51E-19	NS	NS
YE_RS02055	*rpsR*	30S ribosomal protein S18	2.129	2.35E-06	3.445	3.62E-07	2.357	4.01E-07
YE_RS19710	*rpsL*	30S ribosomal protein S12	2.662	5.04E-08	5.235	2.63E-17	2.879	8.99E-09
YE_RS18765	*rpsI*	30S ribosomal protein S9	2.631	2.49E-06	2.333	9.39E-05	2.02	0.000994
YE_RS04260	*rpsP*	30S ribosomal protein S16	2.06	0.000151	4.307	1.74E-13	2.333	1.24E-05
YE_RS19640	*rplP*	50S ribosomal protein L16	NS	NS	3.297	2.24E-12	NS	NS
YE_RS19595	*rplR*	50S ribosomal protein L18	2.412	6.16E-17	4.697	6.46E-36	2.517	2.80E-14
YE_RS19580	*rplO*	50S ribosomal protein L15	2.253	9.31E-18	NS	NS	NS	NS
YE_RS19590	*rpsE*	30S ribosomal protein S5	2.508	5.55E-12	3.492	1.61E-18	NS	NS
YE_RS19560	*rpsK*	30S ribosomal protein S11	2.782	3.92E-11	2.802	1.01E-13	2.051	5.98E-11
YE_RS19565	*rpsM*	30S ribosomal protein S13	2.844	6.46E-14	3.076	9.10E-13	2.354	5.31E-13
YE_RS07755	*rpsA*	30S ribosomal protein S1	NS	NS	2.885	1.72E-05	NS	NS
YE_RS01475	*rplK*	50S ribosomal protein L11	2.378	8.97E-13	7.685	4.76E-30	3.066	1.90E-16
YE_RS19620	*rplX*	50S ribosomal protein L24	2.545	2.01E-09	3.76	5.02E-16	2.085	4.02E-06
YE_RS19625	*rplN*	50S ribosomal protein L14	2.266	3.25E-12	6.21	1.32E-31	3.291	3.80E-23
YE_RS00330	*rpmG*	50S ribosomal protein L33	2.253	2.45E-06	2.664	6.69E-07	NS	NS
YE_RS19555	*rpsD*	30S ribosomal protein S4	3.07	7.26E-20	6.41	3.30E-33	4.723	3.01E-39
YE_RS19670	*rplD*	50S ribosomal protein L4	2.494	1.07E-11	4.296	6.52E-38	2.49	2.36E-14
YE_RS19675	*rplC*	50S ribosomal protein L3	2.894	1.65E-22	5.91	1.89E-29	3.735	2.95E-15
YE_RS02180	*rpmA*	50S ribosomal protein L27	2.117	6.85E-05	2.174	8.77E-05	NS	NS
YE_RS19705	*rpsG*	30S ribosomal protein S7	2.803	2.97E-11	6.297	9.41E-25	3.56	4.08E-17
YE_RS02180	*rpmA*	50S ribosomal protein L27	2.117	6.85E-05	NS	NS	NS	NS

Supplementary Table S4. The growth gap of mutant strain compared with WT in medium at 30 h.

Strains	LBNS	LBNS+R	LBNS+Cys-Cys	LBNS+H	LBNS+REH	LBNS+REHCys-Cys
**Δ*dksA***	17.61%	15.53%	11.79%	13.71%	13.34%	11.87%
**Δ*relA*Δ*spoT***	33.50%	9.11%	10.21%	11.59%	14.79%	18.19%
**Δ*dksA*Δ*relA*Δ*spoT***	32.97%	12.95%	12.98%	12.15%	24.61%	21.11%
**Δ*dksA*(*dksA*)**	10.02%	4.73%	2.05%	-2.48%	-0.18%	-1.92%
**Δ*relA*Δ*spoT*(*spoT*)**	16.29%	10.08%	0.88%	2.94%	3.34%	6.90%

R, Arginine; Cys-Cys, Cystine; H, Histidine; E, Glutamic acid

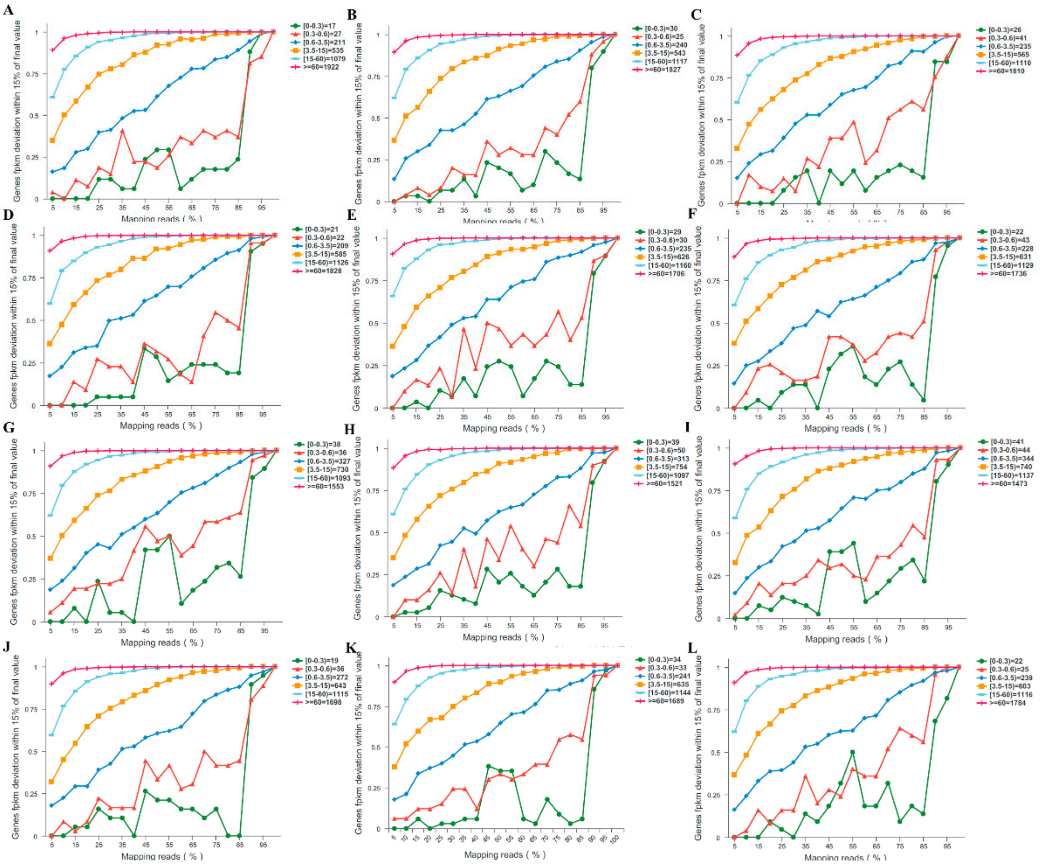

Supplementary Figure S1. Saturation curve of RNA sequencing. (A)-(C) saturation curve of three WT samples; (D)-(F) saturation curve of three Δ*dksA* samples; (G)-(H) saturation curve of three Δ*relA*Δ*spoT* samples; (J)-(K) saturation curve of three Δ*dksA*Δ*relA*Δ*spoT* samples.

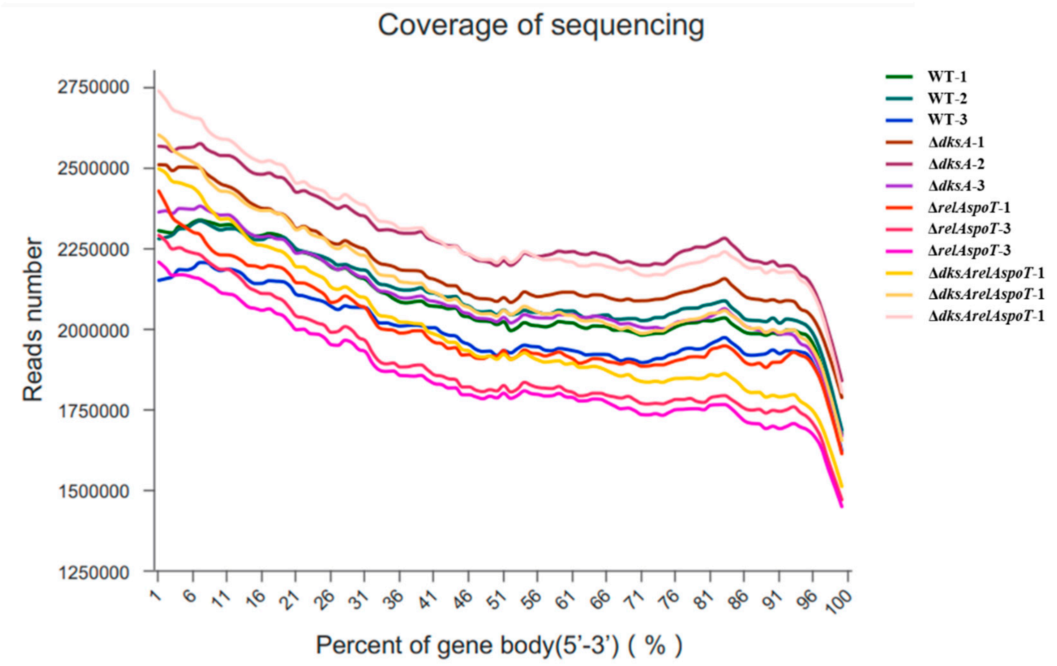

Supplementary Figure S2. The map of sequencing gene coverage.

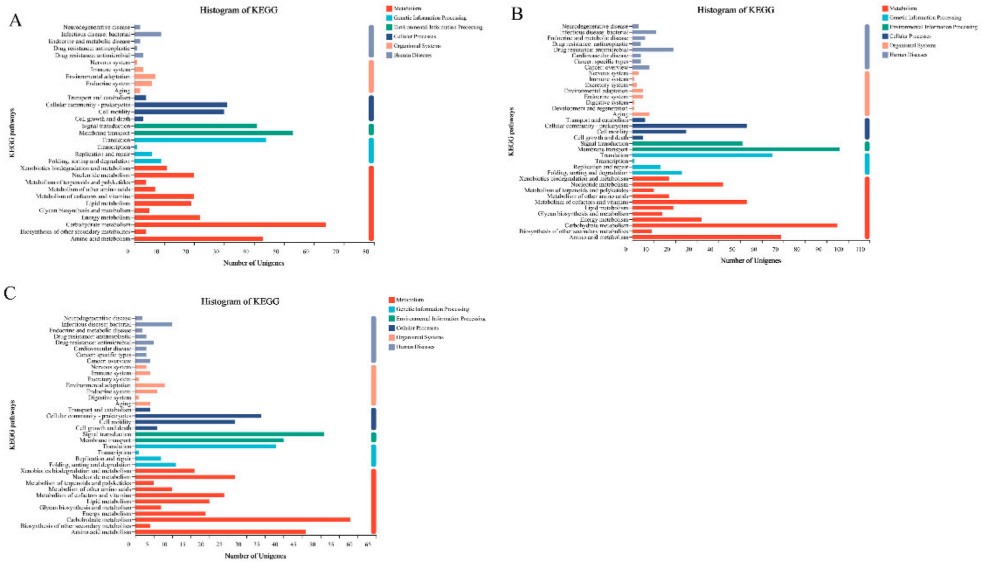

Supplementary Figure S3. Annotation of sequencing results based on KEGG database. (A) Δ*dksA*; (B) Δ*relA*Δ*spoT*; (C) Δ*dksA*Δ*relA*Δ*spoT*.

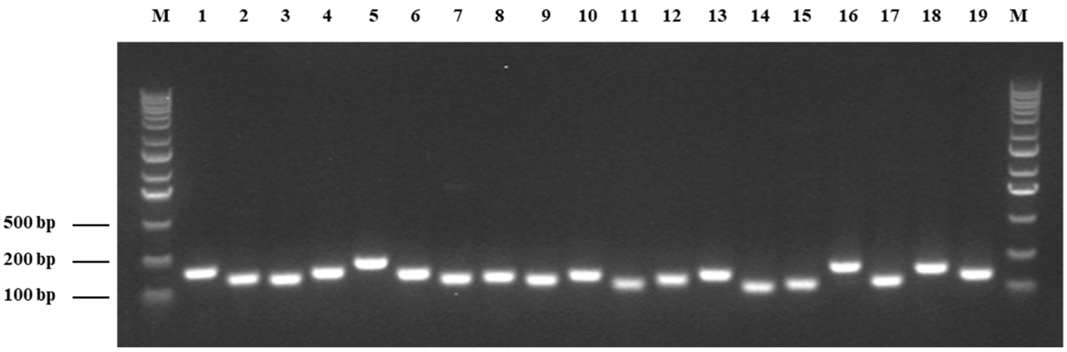

Supplementary Figure S4. Agarose electrophoresis of DNA amplified with the primers used for RT-qPCR. 1-19 represents *pdeR*, *yihX*, *rpsN*, *rpoA*, *trmD*, *dhaK*, *gntT*, *YE_RS15980*, *dusB*, *corA*, *epmA*, *trmL*, *fliE*, *flgC*, *alaC*, *fyuA*, *hypE*, *lsrD*, *hisJ*, respectively.

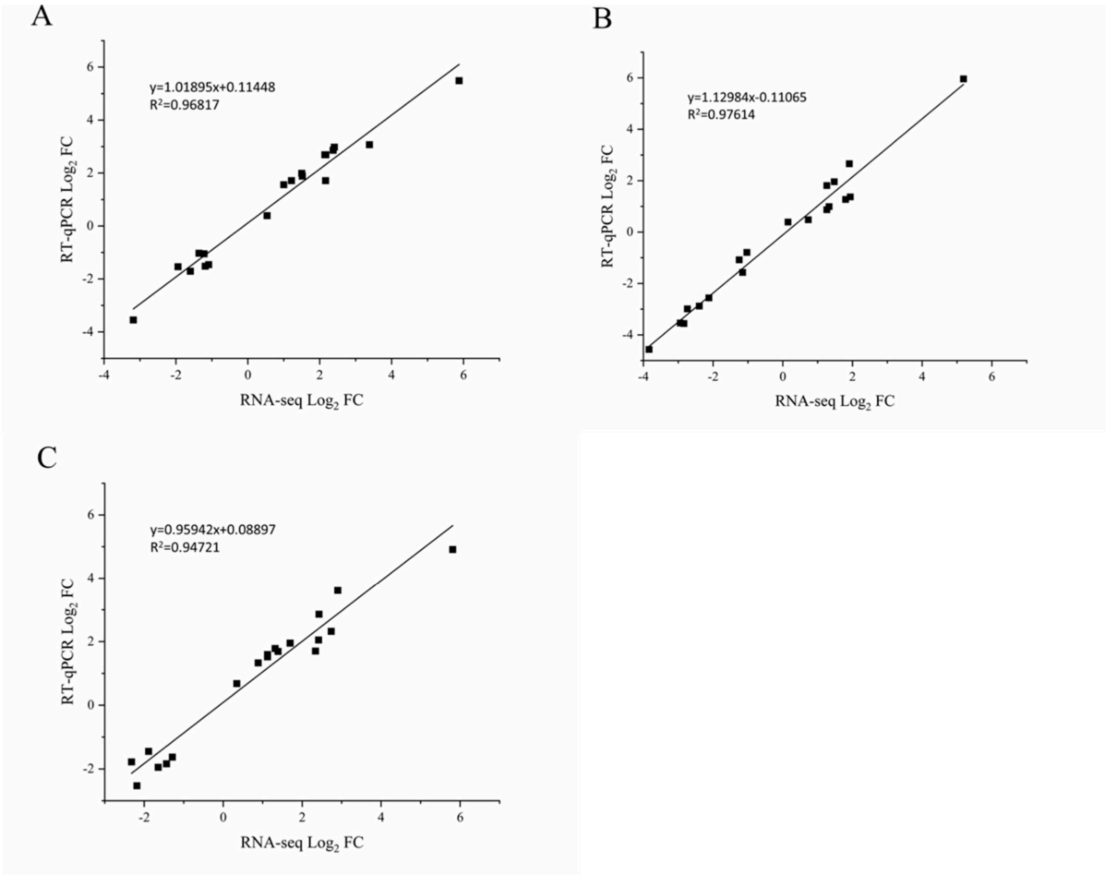

Supplementary Figure S5. Comparison results between RNA-Seq data (X axis) and RT-qPCR data (Y axis). (A) Δ*dksA*; (B) Δ*relA*Δ*spoT*; (C) Δ*dksA*Δ*relA*Δ*spoT*. Data are means and SEM from three independent RT-qPCRs.
